# Role of Omega-3 fatty acids in preventing metabolic disturbances in patients on olanzapine plus either sodium valproate or lithium: a randomized double-blind placebo-controlled trial

**DOI:** 10.1186/2008-2231-20-43

**Published:** 2012-10-04

**Authors:** Toktam Faghihi, Adel Jahed, Javad Mahmoudi-Gharaei, Vandad Sharifi, Shahin Akhondzadeh, Padideh Ghaeli

**Affiliations:** 1 Faculty of Pharmacy, Research Center for Rational Use of Drugs, Tehran University of Medical Sciences, Tehran, Iran; 2 Department of Endocrinology, Booali University Hospital, Islamic Azad University, Tehran Medical Branch, Tehran, Iran; 3 Psychiatry and Psychology Research Center, Roozbeh Hospital, Tehran University of Medical Sciences, Tehran, Iran; 4 Department of Psychiatry, Roozbeh Hospital, Tehran University of Medical Sciences, Tehran, Iran

**Keywords:** Metabolic Disturbance, Hyperlipidemia, Omega-3, Olanzapine, Valproate, Lithium

## Abstract

**Background:**

Metabolic and cardiovascular side effects have been noted with the use of second generation antipsychotics (SGAs) and mood stabilizers. Since Omega-3 fatty acids have been known to prevent some cardiovascular risks, this preliminary study was designed to evaluate the cardiovascular benefits of omega-3 when added to the combinations of olanzapine with mood stabilizers.

**Methods:**

This study was a randomized, double-blind, placebo-controlled, within-subject trial in adult psychiatric patients who were receiving olanzapine combined with lithium (Li) or valproate sodium (VPA). Omega-3 as fish oil with less than 1 g/day of EPA/DHA or its placebo was added to patients’ olanzapine and mood stabilizer regimens for 6 weeks. Metabolic parameters including anthropometric variables, lipid profile, metabolic syndrome indices, C-reactive protein, fibrinogen and lipoprotein (a) [(Lp) (a)] were assessed for participants.

**Results:**

Forty one participants completed this study; 20 patients received omega-3 and 21 patients received placebo, added to their regimen of SGA and mood stabilizer. Omega-3 addition did not modulate anthropometric, metabolic syndrome and lipid parameter changes in 6 weeks. However, fibrinogen levels significantly decreased, Lp (a) did not increase and non-high-density lipoprotein cholesterol (non-HDL-C) did not go beyond its target level after omega-3 supplementation. Additionally, a significant inter-group effect was noted for Lp(a).

**Conclusions:**

This study suggests that use of short-term omega-3 supplementation added to a combined regimen of olanzapine and mood stabilizer may have a small modulating effect on some cardiovascular risk factors. Trials in longer periods of time and with larger number of patients are needed to further evaluate the effects of omega-3 supplements on preventing cardiovascular risk factors.

This trial is registered at irct.ir and its Identifier is as following: IRCT138712231764N1

## Introduction

Second generation antipsychotics (SGAs), in particular clozapine and olanzapine have been linked to excessive weight gain, dyslipidemia, hypertension and development of metabolic syndrome (MetS) [1]. Weight gain is also an unfavorable effect of valproate sodium (VPA) and lithium (Li) [2]. Moreover, VPA has been reported to be associated with dyslipidemia which is independent of weight gain [3]. Psychiatric patients often receive polypharmacy with antipsychotics and mood stabilizers in real practice. It has been noted that patients on polypharmacy regimens have a higher prevalence of metabolic abnormalities [4,5] and more enhanced weight gain [6].

Primary prevention of cardiovascular and metabolic risk development has been suggested to offer the greatest potential to reduce cardiovascular mortality in psychiatric patients [7]. Therefore, it seems mandatory to find safe preventive strategies to attenuate metabolic side effects of psychiatric medications.

Omega-3 polyunsaturated fatty acids in forms of eicosapentaenoic acid (EPA) and docosahexaenoic acid (DHA) in fish oil have been found to correct several cardiovascular risk factors [8-12]. Of note, it has been demonstrated that EPA and DHA can decrease production of inflammatory markers such as IL-1, IL-6 and TNF-α [13]. This may explain the mechanism by which omega-3 fatty acids could be useful in psychiatric patients with a high risk of metabolic abnormalities.

Thus, this preliminary study was designed to evaluate the effects of omega-3 supplements on preventing cardiovascular risk factors by assessing changes in weight, body mass index (BMI), waist circumference (WC), blood pressure (BP) and lipid profiles in a group of psychiatric patients who were on olanzapine plus Li or olanzapine plus VPA for 6 weeks. Additionally, levels of Lp(a), c-reactive protein, high sensitive (CRP-hs) and fibrinogen were also measured in order to explore other cardiovascular risk factors and proinflammatory and procoagulant states in the study subject.

## Materials and methods

This clinical trial with an identifier number of IRCT138712231764N1 was registered at irct.ir and was conducted at Roozbeh hospital, affiliated with Tehran University of Medical Sciences (TUMS). Patients between 18-60 years who were diagnosed with schizophrenia, bipolar I, or schizoaffective disorders according to DSM-IV-TR criteria were included. Subjects were included if they were on combination therapy of olanzapine plus VPA or olanzapine plus Li which was either started simultaneously or the second drug was added within the first 15 days of the initiation of the first medication. This time frame was chosen due to increased prevalence of MetS in patients with bipolar disorder who have been on medications for at least 3 weeks [14].

Exclusion criteria of the study included: (i) treatment with study drugs (olanzapine, VPA, Li) during three months prior to the trial initiation; (ii) any significant medical problem or abnormal laboratory tests before trial participation including: liver function tests ≥ 3 times normal, thyroid stimulating hormone > 4.2 m.I.U/L, GFR < 60 ml/min, fasting blood sugar (FBS) > 125 mg/dl, TG ≥ 500 mg/dl, TG between 200-500 mg/dl with non-high-density lipoprotein cholesterol (non-HDL-C) greater than its target at the time of the study. Non-HDL-C is determined as total cholesterol (TC) minus HDL-C or as very low-density lipoprotein cholesterol (VLDL-C) plus low density lipoprotein cholesterol (LDL-C). It should be mentioned that non-HDL-C treatment goals are defined according to The National Cholesterol Education Program (NCEP) Adult Treatment Panel (ATP) III criteria [15]. Patients were categorized based on a) suffering coronary heart disease (CHD) or CHD risk equivalent, b) not having CHD but having more than 2 risk factors, or c) not suffering CHD and having fewer than 2 risk factors. Treatment goal for each of the above categories if non-HDL-C is greater than 130 mg/dl, 160 mg/dl and 190 mg/dl, respectively); (iii) pregnancy or breastfeeding; (iv) consumption of omega-3 fatty acids recently (within 4 weeks prior to the study initiation); (v) a BMI ≥ 30 kg/m^2^; (vi) any contraindication to receive study drugs; (vii) refusal to participate in the study (by patients or their guardians); (viii) receiving any drug(s), including statins, with much known effects on study parameters; and (ix) a history of any drug or alcohol abuse/dependence within six months prior to the initiation of the study.

The study was performed in accordance with the Declaration of Helsinki and approved by the ethic committee of TUMS. Written informed consent was obtained.

### Study design

This study was designed as a randomized, double-blind, placebo-controlled, within-subject trial in which participants were assigned to receive either omega-3 (fish oil) or placebo. Permuted block randomization table was used and patients were stratified according to participant's smoking status and type of combination therapy (olanzapine plus VPA or olanzapine plus Li). Participants were given omega-3 or identical placebo capsules that were provided by Zahravi Pharmaceutical Company based in Tabriz, Iran for a total of 6 weeks. Each active drug capsule contained 1000 mg omega-3 fatty acids equivalent to 300 mg EPA/DHA. Omega-3 or placebo capsules were titrated up starting with 1 capsule/day in the first week, 2 capsules/day (twice daily) in the second week and 3 capsules/day (in divided doses) from the third week to the end of study. Dose of omega-3 dosage utilized in this study is considered the dietary or low dose supplementation (< 1 g/day EPA/DHA) [16].

Each patient was required to remain stable on his/her combination therapy protocol throughout the course of study (olanzapine plus Li or olanzapine plus VPA); however, dosage adjustment was permitted according to the decisions of the treating psychiatrists. Mean olanzapine, Li and VPA doses were recorded at the trial initiation, during each week and at the end of the study. Other medications that participants may have received at any time during the trial were also recorded (this included both prescription and over-the-counter medications).

Body weight (BW), height, waist circumference (WC) and blood pressure (BP) as well as plasma levels of triglyceride (TG), total cholesterol (TC), low density lipoprotein (LDL), high density lipoprotein (HDL), Lp (a), fibrinogen and CRP-hs were measured at baseline and at week 6. Blood pressure was monitored using mercury sphygmomanometer. BW and WC were assessed in the morning before breakfast. BW was measured without shoes on and in light clothing by a digital scale. WC was determined by placing a measuring tape at the top of right iliac crest and proceeding around the abdomen following normal expiration, ensuring that the tape is tight but not constricting the skin. The "Quetelet Index", BMI was calculated as weight in kilograms (kg) divided by height square in meter square (m^2^). Blood samples were taken between 8 to 9 a.m after a twelve hour overnight fast. Serum TC, HDL, TG, and LDL were measured by enzymatic procedures (LDL was measured directly). Lp (a) and CRP-hs were quantified by immunoturbidimetry and fibrinogen by Clauss coagulation method.

### Statistical analysis

Statistical analysis was carried out with the SPSS statistical package version 15. Demographic characteristics are summarized descriptive and compared using chi-square and independent sample *t*-test statistical method for qualitative and quantitative data respectively. Baseline characteristic in all variables were recorded prior the study and compared using independent sample *t*-test. Paired *t* test were carried out as within group before-after comparison. Comparison of end point changes from baseline was analyzed using within-subjects effects of Repeated Measure ANOVA considering interaction of groups. All statistical significant tests were 2-sided with a nominal significance level 0.05.

## Results

**Figure 1:**
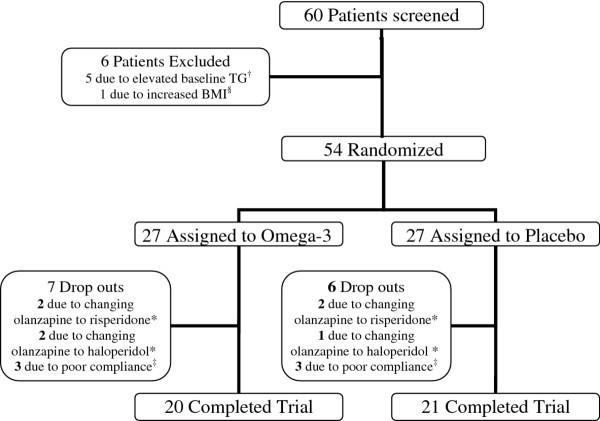
**Trial profile.**^†^TG above 200 mg/dl with determined non-HDL cholesterol above their respective goals. Non-HDL-C treatment goals are defined according to The National Cholesterol Education Program (NCEP) Adult Treatment Panel (ATP) III criteria [15]. Patients were categorized based on a) suffering coronary heart disease (CHD) or CHD risk equivalent, b) not having CHD but having more than 2 risk factors, or c) not suffering CHD and having fewer than 2 risk factors. Treatment goal for each of the above categories if non-HDL-C is greater than 130 mg/dl, 160 mg/dl and 190 mg/dl, respectively). ^§^ BMI ≥ 30 kg/m^2+^. ^‡^ Lack of compliance and discontinuation of psychiatric medications. *Treating physician decision to change olanzapine owing to lack of psychiatric response.

Sixty patients were screened; fifty-four patients were eligible to participate in the study and were randomly assigned to omega-3 and placebo. Forty-one patients completed the 6 week trial and 13 were dropped out during the study (see Figure 1).

There was no statistically significant difference in demographic data at baseline between the two groups (Table 1). The distribution of other medications that participants received during this trial did not differ between the 2 groups (Table 2). There were no statistically significant differences between groups in baseline values of outcome variables, except for fibrinogen level (p = 0.034).

**Table 1:** Demographic characteristic of patients

	**Omega 3**	**Placebo**	**P Value**
	**N = 20**	**N = 21**	
Age (mean ± SD)	31.10 ± 9.98 (year)	36.61 ± 10.92 (year)	0.100
Gender (n)			
Male	14	17	0.484
Female	6	4	
Diagnosis			
Bipolar I disorder	16	13	0.328
Schizophrenia	3	4	
Schizoaffective disorder	1	4	
Lifetime duration of illness (months) (mean ± SD)	27.20 ± 21.29	34.28 ±32.27	0.414

This table has been reproduced from our earlier paper [37] with permission from Iranian Journal of Psychiatry.

**Table 2:** Other medications received by participants

**Medication**	**Number (%) of Participants**	**Placebo**
	**Omega 3**	
**Benzodiazepines**		
Clonazepam	14 (70)	14 (66.6)
Lorazepam	2 (10)	1 (4.76)
Chlordiazepoxide	-	1 (4.76)
**Typical Antipsychotics**		
Haloperidol	12 (60)	13 (61.9)
Chlorpromazine	6 (30)	5 (23.8)
Trifluoperazine	-	1 (4.76)
Flupenthixol Decanoate	1 (5)	1 (4.76)
**Atypical Antipsychotics**		
Risperidone	2 (10)	2 (9.5)
**Other**		
Biperiden	12 (60)	13 (61.9)
Promethazine	2 (10)	2 (9.5)
Hydroxyzine	-	1 (4.76)
Propranolol	4 (20)	5 (23.8)
Trazodone	-	1 (4.76)
Ranitidine	1 (5)	-
Omeprazole	-	1 (4.76)

This table has been reproduced from our earlier paper [37] with permission from Iranian Journal of Psychiatry.

At the end point, mean daily dosage of olanzapine was 13.00 ± 4.97 mg in the omega-3 and 12.85 ± 4.35 mg in the placebo group (p = 0.922). VPA mean dosage in omega-3 and placebo group were 749.00 ±189.73 and 608.33 ± 235.32 mg (p = 0.170), respectively. On the other hand, Li mean dosage in omega-3 and placebo group were 1057.50 ± 288.20 and 1150.00 ± 300.00 mg (p = 0.502), respectively.

At the end of this trial, the following observations were noted. Mean weight, BMI and WC increased significantly in both groups. There were no statistically significant changes in systolic and diastolic BP within each group. TC, LDL and non-HDL-C increased significantly in both omega-3 and placebo groups. There was no significant change in TG in any of the groups. HDL did not change in omega-3 group but significantly increased in the placebo group. Lp (a) did not significantly change in the omega-3 group but significantly increased in the group receiving placebo added to their regimen of olanzapine with Li or VPA. Additionally, a significant inter-group effect was noted for Lp(a). Fibrinogen levels significantly decreased in omega-3 group but its decrease in placebo group was not statistically significant. There was no statistically significant change in CRP-hs level within each group (Table 3). Generally, omega-3 was well tolerated by patients in this study. Mild fishy odor was the only complaint that was expressed by one of the participants.

**Table 3:** Study Measures at baseline and at the end point

**Variables**	**Omega 3**		P^*^	**Placebo**		P^*^	**Group effect comparison**	
	**Baseline**	**End point**		**Baseline**	**End point**		**F**	P^**^
	**Mean(SD)**	**Mean(SD)**		**Mean(SD)**	**Mean(SD)**			
BW† (kg)	70.68 ± 13.35	75.03 ± 13.98	0 < 001	74.27 ± 11.17	78.35 ± 14.55	0.008	0.025	0.875
BMI (kg/m^2^)	24.43 ± 3.03	25.95 ± 3.27	0 < 001	24.95 ± 3.63	26.28 ± 4.66	0.004	0.137	0.714
WC (cm)	83.59 ± 8.88	86.84 ± 8.94	0.007	88.13 ± 13.23	91.02 ± 13.48	0.003	0.071	0.791
SBP (mmHg)	119.50 ± 9.98	115.50 ± 11.45	0.088	113.80 ± 9.34	115.50 ± 8.21	0.560	3.011	0.091
TC (mg/dl)	172.30 ± 34.94	187.90 ± 34.55	0.023	177.66 ± 37.78	206.09 ± 52.71	0.001	1.69	0.201
LDL (mg/dl)	86.40 ± 22.16	100.30 ± 17.93	0.004	91.09 ± 24.49	104.14 ± 30.78	0.004	0.021	0.885
HDL (mg/dl)	35.45 ± 9.04	35.45 ± 8.78	1.000	37.23 ± 11.30	39.23 ± 10.21	0.021	0.868	0.357
Non-HDL-C (mg/dl)	136.85 ± 30.63	152.45 ± 30.29	0.013	141.28 ± 34.73	166.85 ± 50.83	0.001	1.387	0.246
TG (mg/dl)	162.90 ± 72.51	165.95 ± 68.79	0.825	168.90 ± 71.83	171.76 ± 88.54	0.865	0.000	0.993
Lipo (a) (mg/dl)	46.64 ± 43.52	43.85 ± 29.81	0.623	45.35 ± 38.21	65.47 ± 57.52	0.042	4.507	0.040
Fibrinigen (mg/dl)	321.95 ± 56.41	283.80 ± 71.62	0.041	286.23 ± 47.34	282.57 ± 63.83	0.787	2.497	0.122
CRP-hs (mg/dl)	4.33 ± 4.198	4.11 ± 8.46	0.969	3.10 ± 3.17	3.49 ± 3.03	0.634	0.017	0.897

† BW, body weight; BMI, body mass index; WC, waist circumference; SBP, systolic blood pressure; TC, total cholesterol; LDL, low-density lipoprotein; HDL, high-density lipoprotein; Non-HDL-C, non high-density lipoprotein-cholesterol; TG, triglyceride; Lipo (a), lipoprotein Lp(a); CRP-hs, c-reactive protein, high sensitivity.

* P-values are given for the comparison of before-after changes in each group using paired-samples *t* test.

** P-values are given for comparison of end point changes from baseline using within-subjects effects of Repeated Measure ANOVA considering interaction of groups.

## Discussion

In this study, we evaluated the probable benefits of dietary dose of omega-3 fatty acids on some traditional and non-traditional cardiovascular risk factors, when added at the beginning of SGAs and mood stabilizers before the establishment of the metabolic side effects.

In this study, we did not observe a change in BP. Fish oil (containing omega-3) supplementation for a median duration of eight weeks has been shown to lower systolic and diastolic blood pressures [9]. BP reduction pattern of fish oils is also shown to be linear at dietary doses (EPA plus DHA <1 g/day) and reaches plateaus at higher than the dietary doses [16]. This effect is also claimed to be less pronounced in healthy adults younger than 45 years-old [9,16] and may take a longer time to take place [16]. Thus, the unchanged BP in this study may be attributed to the younger age of the participants and the short duration of trial.

Omega-3 fatty acids did not favorably affect LDL and TC levels in our study; however, a trend toward lower TC increment in the omega-3 group (15.6 mg/dl) than that in placebo group (28.43 mg/dl) may suggest that omega-3 may prevent increases in TC levels. Studies have shown that omega-3 fatty acids can decrease small dense LDL, a highly atherogenic form of LDL [17]. Thus, it is suggested to measure this form of LDL when studying effects of omega-3 on cholesterol levels.

With regard to HDL, McKenney and Sica reported that omega-3 fatty acids can only modestly increase this factor [18]. This may partly explain the observed unchanged values of HDL in our study. Contrary to our expectations, HDL levels were increased significantly in the placebo group that may be related to the fact that our sample size was not sufficient to detect more pronounced effects for some of the outcome variables including HDL.

Despite our hypothesis, omega-3 fatty acids did not reduce TG levels in our study. Some studies have shown that TG lowering effects of omega-3 fatty acids occur within months to years and with a greater effect in patients with higher baseline TG levels [16,17]. In addition, effect of omega-3 on lowering TG concentration appears to be linear; omega-3 in doses of 3-4 g/day have greater effect compared with doses <1g/day [16]. Therefore, the reason that TG levels were not reduced in our study may be due to the fact that psychiatric patients without important medical problems were included, lower EPA and DHA doses were utilized, baseline TG levels were not considered in the high range and this trial was performed in a relatively short duration of time.

Current guidelines identify non-HDL-C as a secondary target of therapy in patients with TG ≥ 200 mg/dl after achieving LDL goals [15]. Non-HDL-C increased significantly in each study group; however, no difference was noted between the two groups. Interestingly, when evaluating patients whose TG levels were above 200 mg/dl at the end of the present trial, increase in non-HDL-C, requiring treatment, was identified in 2.4% of participants in the omega-3 group versus 14.6% in the placebo group. This is while, at the beginning of the study, we excluded patients with non-HDL-C levels requiring treatment according to NCEP ATP III guidelines [15], from entering the study. Thus, the above finding may suggest a promising role for dietary doses of omega-3 to prevent increases of non-HDL-C concentrations above target levels in patients receiving psychiatric medications with metabolic side effects.

Along with elevated LDL cholesterol, elevated Lp(a) is considered to promote atherosclerosis [19]. Lp(a) serum levels are primarily, genetically controlled, however, its levels may also be affected by some exogenous factors [20]. Long term treatment with anti-epileptic drugs, specifically VPA is shown to increase Lp(a) levels in patients with epilepsy [20-23]. Currently, medication effects on Lp(a) are limited. According to the results of our study, omega-3 supplementation prevented Lp(a) elevation due to therapy. In line with our results, a favorable effect of omega-3 on decreasing Lp(a) serum levels was previously demonstrated in hypertensive and coronary artery disease patients [24,25].

MetS is a procoagulant state with elevated fibrinogen level as a contributor. Elevated fibrinogen has been observed in patients with obesity [26]. Additionally, increased fibrinogen plasma levels are considered a risk factor for major CVDs including coronary heart disease and stroke [27]. Baptista and his colleagues hypothesized that olanzapine increases fibrinogen levels, leading to a procoagulant state [26]. Another study demonstrated elevated levels of fibrinogen after 12 weeks of olanzapine administration in patients with schizophrenia [28]. In contrast to olanzapine, VPA has reported to reduce fibrinogen levels [23,29,30], indicating impaired hepatic synthetic function [29]. The above studies did not assess the relevance of decreased fibrinogen levels to CVD risk. However, VPA may still lead to atherosclerosis and thrombosis despite decreased fibrinogen levels [30]. In our study fibrinogen plasma levels did not change in the group assigned to placebo. Even although the effects of most of the other medications on this parameter is yet unclear, the above finding may reflect a probable combined effect of olanzapine and valproate on fibrinogen plasma levels. Interestingly, in the group assigned to omega-3, fibrinogen levels decreased significantly by 38.15 mg/dl (from 321.95 mg/dl to 283.80 mg/dl). The normal range of fibrinogen level is 200-400 mg/dl [31]. Whether this decrease in fibrinogen level corresponds to CVD reduction, remains to be elucidated.

Evidence supports inflammatory processes contributing to atherogenesis. Centers for Disease Control and Prevention and the American Heart Association have defined values of CRP-hs <1, 1-3 and > 3 mg/dl as low, average and high risks to develop CVD, respectively [32]. It should be noted that elevated levels of CRP has been observed in patients with BD and schizophrenia which may suggest an inflammatory component in these psychiatric illnesses [33,34]. At baseline we observed CRP-hs values of 4.33 and 3.1 mg/dl in the omega-3 and placebo groups, respectively; these values remained elevated throughout the study period. The above observation suggests an inflammatory process occurring in psychiatric patients and remaining beyond the acute phase of the involved illnesses. This is consistent with Dickerson et al. report on elevated levels of CRP in outpatients with a relatively long duration of BD. Our results also showed that omega-3 fatty acids did not have any significant effect on CRP-hs levels. In general, controlled trials have not detected significant effects of omega-3 supplementation on CRP-hs levels [35].

Despite the suggested protective role of omega-3 against abdominal obesity [36], this supplement could not lessen increases in weight and WC in our study patients. After 6 weeks, our participants significantly gained a mean of 4.35 kg in the omega-3 group and 4.07 kg in the placebo group. Few studies have reported the extent to which each specific combination therapies of Li or VPA plus olanzapine increase weight. This study was not designed to assess weight changes related to various combination therapies or to any of these agents alone. However, our results were similar to those reported by Kim et al. who reported weight gain of 3.8 ± 2.9 kg with olanzapine plus VPA and 3.3 ± 3.1 kg with olanzapine plus Li. Kim et al. also found that olanzapine monotherapy resulted in 1.6 ± 3.5 kg increase in weight after 4 weeks in patients with BD [6].

It should be emphasized that the short duration of our study set limit to observe more profound effects. Additionally, even though the assessment of omega-3 effects was aimed to be accomplished in a realistic setting, receiving combination of olanzapine with a mood stabilizer by patients might have influenced the results of the present study. Another limitation that could influence our study was the fact that even though patients were asked not to change their regular diet during the study, they were not strictly controlled for their diet calories taken and activity levels.

In conclusion the addition of dietary doses of omega-3 fatty acid supplements per day at the commencement of olanzapine combination with either Li or VPA may not prevent or modulate early anthropometric and lipid profile deterioration. However, this study noted that Lp(a) level was not elevated and fibrinogen level was decreased in patients receiving omega-3 fatty acids for six weeks. A trend toward smaller increases in TC levels and the ability to prevent non-HDL-C exceeding its target in patients with TG levels above 200 mg/dl supports some cardiovascular benefit of low dose omega-3.

## Conclusion

This study suggests that short-term use of omega-3 supplementation added at the beginning of a combined regimen of olanzapine and mood stabilizer may only benefit by modulating some non-traditional cardiovascular risk factors. Trials in longer periods of time and with larger number of patients are recommended to further evaluate the effects of omega-3 supplements on preventing cardiovascular risk factors.

## Competing interests

The authors declare that they have no competing interests.

## Authors’ contribution

FT main researcher (this manuscript was a big part of Dr Faghihi’s post-Pharm.D residency thesis) who was involved in all aspects of the project. JA endocrinologist who was involved in designing the trial, interpretation of the data, and giving comments regarding the manuscript before its submission. MJ psychiatrist who was involved in designing the trial and analysis of the data. SV psychiatrist who was involved in designing the trial and verifying the diagnosis of the patients. AS Neuroscientist and psychopharmacologist involved in approving the scientific basis of the trial and consultation regarding different steps of the trial. GP clinical Psychiatric Pharmacist who was the main supervisor of this project and was involved in all aspects of the study and the corresponding author. All authors read and approved the final manuscript.
